# A Pathway-Based Genetic Score for Oxidative Stress: An Indicator of Host Vulnerability to Phthalate-Associated Adverse Neurodevelopment

**DOI:** 10.3390/antiox11040659

**Published:** 2022-03-29

**Authors:** Samuel Tanner, Sarah Thomson, Katherine Drummond, Martin O’Hely, Christos Symeonides, Toby Mansell, Richard Saffery, Peter D. Sly, Fiona Collier, David Burgner, Eva J. Sugeng, Terence Dwyer, Peter Vuillermin, Anne-Louise Ponsonby, on behalf of the Barwon Infant Study Investigator Group

**Affiliations:** 1 Developing Brain Division, The Florey Institute for Neuroscience and Mental Health, Parkville, VIC 3052, Australia; sam.tanner@florey.edu.au (S.T.); sarah.thomson@florey.edu.au (S.T.); katie.drummond@unimelb.edu.au (K.D.); 2 Murdoch Children’s Research Institute, Royal Children’s Hospital, University of Melbourne, Parkville, VIC 3052, Australia; martin.ohely@deakin.edu.au (M.O.); christos.symeonides@mcri.edu.au (C.S.); toby.mansell@mcri.edu.au (T.M.); richard.saffery@mcri.edu.au (R.S.); fmcol@deakin.edu.au (F.C.); david.burgner@mcri.edu.au (D.B.); terence.dwyer@wrh.ox.ac.uk (T.D.); peter.vuillermin@deakin.edu.au (P.V.); 3 School of Medicine, Deakin University, Geelong, VIC 3216, Australia; 4 The Minderoo Foundation, Perth, WA 6000, Australia; 5 Children’s Health Research Centre, University of Queensland, South Brisbane, QLD 4101, Australia; p.sly@uq.edu.au; 6 WHO Collaborating Centre for Children’s Health and Environment, South Brisbane, QLD 4104, Australia; 7 Barwon Health, Geelong, VIC 3216, Australia; 8 Department of Paediatrics, University of Melbourne, Parkville, VIC 3052, Australia; 9 Department of Environment and Health, Vrije Universiteit, De Boelelaan 1087, 1081 HV Amsterdam, The Netherlands; e.j.sugeng@vu.nl; 10 Nuffield Department of Women’s & Reproductive Health, University of Oxford, Oxford OX3 9DU, UK

**Keywords:** oxidative stress, biological pathway, genetic score, neurodevelopment, cognition, autism, ASD, attention-deficit hyperactivity disorder, ADHD, plastics, phthalates

## Abstract

The developing brain is highly sensitive to environmental disturbances, and adverse exposures can act through oxidative stress. Given that oxidative stress susceptibility is determined partly by genetics, multiple studies have employed genetic scores to explore the role of oxidative stress in human disease. However, traditional approaches to genetic score construction face a range of challenges, including a lack of interpretability, bias towards the disease outcome, and often overfitting to the study they were derived on. Here, we develop an alternative strategy by first generating a genetic pathway function score for oxidative stress (gPFS^ox^) based on the transcriptional activity levels of the oxidative stress response pathway in brain and other tissue types. Then, in the Barwon Infant Study (BIS), a population-based birth cohort (n = 1074), we show that a high gPFS^ox^, indicating reduced ability to counter oxidative stress, is linked to higher autism spectrum disorder risk and higher parent-reported autistic traits at age 4 years, with AOR values (per 2 additional pro-oxidant alleles) of 2.10 (95% CI (1.12, 4.11); *p* = 0.024) and 1.42 (95% CI (1.02, 2.01); *p* = 0.041), respectively. Past work in BIS has reported higher prenatal phthalate exposure at 36 weeks of gestation associated with offspring autism spectrum disorder. In this study, we examine combined effects and show a consistent pattern of increased neurodevelopmental problems for individuals with both a high gPFS^ox^ and high prenatal phthalate exposure across a range of outcomes, including high gPFS^ox^ and high DEHP levels against autism spectrum disorder (attributable proportion due to interaction 0.89; 95% CI (0.62, 1.16); *p* < 0.0001). The results highlight the utility of this novel functional genetic score and add to the growing evidence implicating gestational phthalate exposure in adverse neurodevelopment.

## 1. Introduction

Disorders of child neurodevelopment have increased over recent decades. Since the 2000s, for instance, the prevalence of autism spectrum disorder (ASD) has risen from 6.7 in 1000 [1] to 17.2 in 1000 in the United States [2], with a global estimate of 1–2.5% [3]. Reported prevalence rates of developmental delay in childhood have also risen [4,5]. The increases may partly reflect heightened awareness, improved detection, and changes in diagnostic criteria [6]. However, given the sensitivity of the developing brain to environmental disturbances [7], there is growing concern around the role of prenatal chemical exposures, including exposure to manufactured plastic chemicals [8,9,10]. 

Phthalates are one chemical class of concern [11,12]. They are a ubiquitous environmental contaminant [13] and can pass through the placental barrier and affect the developing fetus [14]. We have previously reported that the majority of pregnant women in our location harbor phthalate chemicals [15]. These chemicals appear to derive from ingested food (plastic food packaging, tinned food, and high-fat milk) and inhaled (air freshener and aerosols) and absorbed sources (hair treatment and cleaning chemicals) [15]. This is consistent with previous reports [16,17,18,19,20,21], which have highlighted plastic food packaging in particular as a major phthalate source. 

Epidemiological studies have overall reported a positive association between prenatal phthalate exposure and adverse neurodevelopment [22], as described in recent systematic reviews [23,24,25,26], although not all findings have been consistent. There is some evidence for a possible adverse effect on child cognitive development, with maternal urinary phthalate metabolites linked to lower cognition [23,26]. Prenatal phthalate exposure is also associated in some studies with increased ASD symptomology and diagnosis risk [27,28,29,30,31], including in our location [31]. We have suggested that disparities may, in part, be attributable to differing susceptibility to toxicants, due to genetics and concomitant oxidant exposures [31]. In the Barwon Infant Study cohort, we reported that an infant’s genetic vulnerability to oxidative stress, an imbalance between the production and detoxification of reactive oxygen species (ROS) [32], may interplay with maternal phthalate exposure to increase the risk of adverse neurodevelopment [31].

In other studies of human cohorts and preclinical animal and cell culture models, phthalates have been linked to elevated oxidative stress [33,34,35]. A consequent impact on neurodevelopment is plausible as the brain’s high oxygen requirements make it especially susceptible to the damaging effects of ROS. Excess ROS can impair neurodevelopment by suppressing neuronal proliferation and migration, synapse formation, and long-term potentiation, and by activating inflammatory and apoptotic pathways in the brain [36]. Indeed, genome-wide association studies (GWAS) and other genomic analyses of ASD routinely identify genes implicated in oxidative stress [37,38,39,40].

Given that the ability of an individual to respond effectively to oxidative stress is determined in part by genetic makeup, several studies of ROS-related disorders have used polygenic risk scores (PRSs) to quantify genetic predisposition to oxidative stress [41,42]. A PRS is computed by summing the number of single-nucleotide polymorphisms (SNPs) an individual carries that show association with the trait or disease of interest, with each SNP often weighted by its GWAS effect size [43,44]. 

However, standard approaches to genetic score construction, including for oxidative stress, face a range of challenges. A key limitation is that the SNPs employed in PRSs rarely map to functionally coherent gene sets, making such scores difficult to interpret mechanistically [45]. Further, some oxidative-stress-related PRSs encode a disease outcome as well as oxidative stress, creating a bias towards this outcome that strengthens associations but obscures the impact of oxidative stress, per se, on the disease. For instance, we have previously published on a genetic score for oxidative stress derived from SNPs in genes relevant to oxidant balance that were further filtered based on association with adverse neurodevelopment [31]. Moreover, these challenges are frequently compounded by overfitting due to scores being both generated and evaluated on the same study [46,47].

Here, we present an alternative approach using a genetic pathway function score for oxidative stress (gPFS^ox^), constructed independently of our study cohort, and capturing transcriptional activity of the oxidative stress response pathway. This biologically motivated, disease-agnostic score has the potential to be more interpretable and applicable to a wider range of brain and other disorders than existing PRSs. By encoding activity at the pathway level, it can help elucidate how the *mechanism* of ROS production and elimination impinges on neurodevelopment. 

In this study, we aim (i) to examine the association between this gPFS^ox^ score and key neurodevelopmental outcomes relating to cognition and attention-deficit hyperactivity disorder at 2 years of age and autism at ages 2 and 4 years in a large population-based cohort, and (ii) to investigate the interplay between gPFS^ox^ and prenatal phthalate exposure in adverse neurodevelopment. We discuss how the findings compare to our past use of a disease-based genetic score for the same study [31].

## 2. Materials and Methods

### 2.1. Study Cohort

The Barwon Infant Study (BIS) is a birth cohort from Victoria, Australia, consisting of 1074 mother-infant pairs and aimed at investigating early-life causes of non-communicable diseases [48]. Women were recruited between 15 and 28 weeks of completed pregnancy from 2010 to 2013 but later excluded if their child was born before 32 weeks or diagnosed with a congenital disorder or serious illness. As described elsewhere, extensive biological, clinical, and questionnaire measures were collected prenatally, at birth, and in intervals up to age 4 years [48]. The study was approved by the Barwon Health Human Research Ethics Committee (HREC 10/24) and written informed consent was obtained from the participating families.

### 2.2. Phthalate Measurement

Phthalate metabolite levels were measured in 842 pregnant women using a single spot urine specimen collected at 36 weeks of gestation. High-performance liquid chromatography–tandem mass spectroscopy with direct injection was performed by the Queensland Alliance for Environmental Health Science (QAEHS). QAEHS procedures are detailed elsewhere [49]. For monoethyl phthalate (MEP), monoisobutyl phthalate (MiBP), mono-n-butyl phthalate (MnBP), mono-(2-ethyl-5-hydroxyhexyl) phthalate (MEHHP), and mono-(2-ethyl-5-oxohexyl) phthalate (MEOHP), repeated spot specimens taken in the third trimester had intra-class correlation coefficients over 0.4 in one or both of two previous studies [50,51]. This suggests that single spot tests can capture third-trimester exposure to phthalates with reasonable reliability.

### 2.3. Child Neurodevelopmental Outcomes

At age 2–3 years, the child’s caregiver completed the Child Behavior Checklist for Ages 1.5–5. The DSM-5-oriented autism spectrum problems (CBCL-ASP) and attention-deficit hyperactivity disorder (CBCL-ADHD) subscales were derived by summing responses to 12 behavioral statements (0: not true; 1: somewhat true; 2: very true) [52]. A scaled T-score of 50 represents the median in the normative sample, so a binary variable was created whereby children with a T-score above 50 were classified as having “above-median CBCL autism spectrum problems”. For CBCL-ADHD, a binary variable was created whereby for children with a T-score above 65, the threshold for “borderline or clinical ADHD symptoms” [53] was classified as having “high-CBCL attention-deficit hyperactivity problems”. At age 2–3 years, the Bayley Scales of Infant and Toddler Development 3rd edition (BAYLEY-III) cognitive scale was administered, and the raw scores were used in analyses [54]. At age 4 years, parents reported if their child had doctor-diagnosed autism spectrum disorder (ASD) or any ASD traits. 

### 2.4. Genotyping

DNA samples were extracted from cord and 12-month whole blood using the QIAamp 96 DNA QIAcube HT kit (QIAGEN, Hilden, Germany) according to manufacturer’s instructions and stored at −80 °C. Whole-genome genotyping was performed with an Illumina Global Screening Array (Illumina, San Diego, CA, USA). Imputation was completed using the Sanger Imputation Server (Wellcome Sanger Institute, Hinxton, UK) based on the Haplotype Reference Consortium reference panel [55,56]. Infants were excluded for quality control if initial genotyping was unsuccessful at more than 5% of SNPs, and SNPs were dropped if (i) genotyping failed across more than 5% of infants, (ii) minor allele frequency was less than 0.01 or differed by more than 0.2 from the reference population, or (iii) the SNPs were not in Hardy–Weinberg equilibrium [31].

### 2.5. Genetic Pathway Function Score for Oxidative Stress

**Figure 1:**
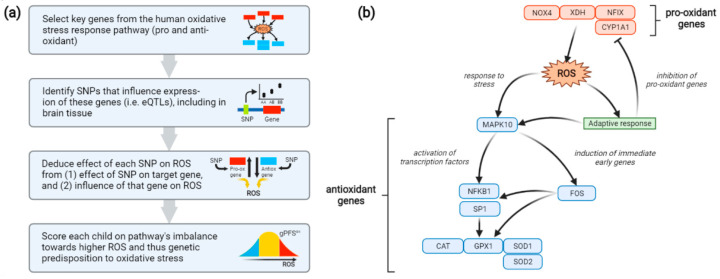
(**a**) Method used to construct a genetic pathway function score for oxidative stress (gPFS^ox^). (**b**) Oxidative-stress pathway encoded using gPFS^ox^. Included are 4 genes involved in the production of ROS (red) and 8 genes involved in the response to ROS (blue). *NOX4*, NAD(P)H oxidase 4; *XDH*, xanthine dehydrogenase; *NFIX*, nuclear factor I X; *CYP1A1*, cytochrome P450 family 1 subfamily A member 1; *MAPK10*, mitogen-activated protein kinase 10; *NFKB1*, nuclear factor kappa B subunit 1; *SP1*, specificity protein 1; *CAT*, catalase; *GPX1*, glutathione peroxidase 1; *SOD1*, superoxide dismutase 1; *SOD2*, superoxide dismutase 2; ROS, reactive oxygen species; SNP, single-nucleotide polymorphism; eQTL, expression quantitative trait loci.

To capture each participant’s genetic predisposition to oxidative stress, a genetic pathway function score (gPFS^ox^) was generated as follows (Figure 1a). First, a minimal pathway for the human oxidative stress response was constructed, consisting of 4 pro-oxidant and 8 antioxidant genes acting in opposing directions (Figure 1b) [57,58,59,60].

Single-nucleotide genetic polymorphisms (SNPs) linked to the activity of these genes were then identified using the Genotype-Tissue Expression (GTEx) database [61,62]. GTEx provides information on SNPs associated with tissue-specific expression of query genes by leveraging genotyping and RNA sequencing data across 54 tissue types from nearly 1000 individuals. For each oxidative stress gene, the SNP most strongly associated with its expression in any tissue type was chosen. These SNPs showed generally consistent effects across multiple tissue types, including the brain. The influence of each SNP on oxidative stress was then deduced from two factors: (1) whether the SNP up- or down-regulates expression of its target gene, and (2) whether the target gene elevates or reduces oxidative stress (Table 1). For instance, a pro-oxidant SNP might up-regulate expression of a pro-oxidant gene or down-regulate expression of an antioxidant gene. Finally, a score was created for each BIS participant reflecting the number of pro-oxidant alleles they carry for the oxidative stress response pathway and hence the pathway’s cumulative imbalance, at the transcriptional level, towards oxidative stress. Thus, if the *p* SNPs are ordered such that SNPs 1, …, *p_pro_* are pro-oxidant and SNPs *p_pro_* + 1, …, *p* are antioxidant, the score is computed as:

$${\mathrm{gPFS}}_{i}^{\mathrm{ox}}=\frac{1}{2}\left(\overset{p_{pro}}{\underset{j=1}{\sum }}C_{j}+\overset{p}{\underset{j=p_{pro}+1}{\sum }}\left(2-C_{j}\right)\right)$$

where gPFS*_i_*^ox^ is the score for individual *i*, *j* is the current SNP out of *p* = 12 total SNPs across the pathway (one for each gene), and *C* is the allele count for SNP *j* (0, 1 or 2). Thus, for each SNP, the count of pro-oxidant alleles at that locus is added; for a pro-oxidant SNP, this is simply the number of effect alleles present, while for an antioxidant SNP it becomes 2 minus the number of effect alleles. For convenience, the score is then divided by 2 to range from 0 to 12 units, the number of genes in the pathway, rather than 0 to 24 units, the number of alleles for the 12 corresponding SNPs. 

### 2.6. Statistical Analysis

Phthalate metabolite measurements were corrected for processing batch, urine dilution, and time of day for spot sample collection [31]. To estimate the biological dose delivered to the fetus, phthalate daily intakes were calculated accounting for maternal prenatal weight, average daily urine volume, fractional excretion of the compound, and compound-to-metabolite molecular weight ratio [31]. The metabolites MEHHP, MEOHP, and mono-(2-ethyl-5-carboxypentyl) phthalate (MECPP) were used to calculate di-(2-ethylhexyl) phthalate (DEHP) daily intake; MEP for diethyl phthalate (DEP); MiBP for diisobutyl phthalate (DiBP); and MnBP for di-n-butyl phthalate (DnBP). DiBP and DnBP daily intakes were summed to make a DBPs daily intake measure and were not considered individually in the following analyses. DEHP, DEP, DiBP, and DnBP daily intakes were summed to make a composite phthalate daily intake measure (Σ phthalates).

Multiple linear regression was used to test for association between gPFS^ox^ and Bayley-III cognition, a continuous measure of cognitive ability. For binary outcomes, multiple logistic regression was used; these were high-CBCL attention-deficit hyperactivity problems, above-median CBCL autism spectrum problems, ASD diagnosis, and ASD traits. 

Interplay with prenatal phthalate exposure was assessed by (i) evaluating the risk of adverse neurodevelopment associated with top quintile of gPFS^ox^ and top quintile of phthalate levels vs. bottom four quintiles for both and (ii) testing for additive interaction. We present the attributable proportion (AP), which measures the proportion of the disease in the doubly exposed group that is due to the interaction [63] and the relative excess risk due to interaction (RERI), a measure of interaction on the additive scale for risk ratios [64].

For (i), G^hi^ or P^hi^ indicated children with values in the top quintile for gPFS^ox^ or phthalates respectively, whereas children classified as G^lo^ or P^lo^ were in the lower 4 quintiles for gPFS^ox^ or phthalates, respectively. 

For the cognition outcome, adjustment was made for post-conceptional age at testing, sex, administering researcher, and experience of researcher in test administration. For high-CBCL attention-deficit hyperactivity problems, above-median CBCL autism spectrum problems, ASD diagnosis, and ASD traits, adjustment was made for post-conceptional age at testing and sex. A *p*-value threshold of 0.05 was used for statistical significance. All analyses were conducted in R 4.0.0 (R Foundation for Statistical Computing, Vienna, Austria) and Stata/SE 16.1 (Stata Statistical Software, College Station, TX, USA).

## 3. Results

**Figure 2:**
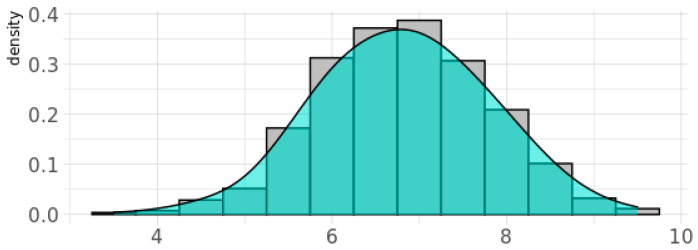
Density plot for the genetic pathway function score for oxidative stress response (gPFS^ox^) in the BIS cohort. Note: Y-axis gives the probability density function for the kernel density estimation used to fit the curve.

The genetic score for oxidative stress, gPFS^ox^, was normally distributed for BIS children, with 20% of children having a value of 8 units or above (Figure 2). Children in this category were classified as G^hi^ (i.e., showing greater genetic vulnerability to oxidant damage due to reduced antioxidant defenses), while those below were classified as G^lo^. Of the 842 women with phthalate measurements in the inception cohort, the percentage with detectable levels of metabolites of DEHP was 100%, for DnBP and DEP was 99%, and for DiBP was 98%, and these levels varied by more than 1000-fold [31]. For each phthalate daily intake measure, children in the top quintile (i.e., those with the highest prenatal exposure levels) were classified as P^hi^ and children in the bottom four quintiles as P^lo^. At age two years, 37% of children (55% male) had ASD symptoms classified as above average according to the CBCL-ASP scale (Table 2). At age four years, 1.4% (11/791) of children (55% male) were reported to be diagnosed with ASD, most of which were verified (9/11, 82%) in pediatric medical records, while 4.9% (39/791; 62% male) were reported to have ASD traits (Table 2). A continuous CBCL ASP scale at the age of 2 years predicted later ASD diagnosis at the age of 4 years with an area under the curve (AUC) score of 0.93 (95% CI (0.82, 1.00)) [65].

### 3.1. Association between Phthalate Daily Intake and Neurodevelopmental Outcomes

As previously reported [31], prenatal daily intake of DBPs, DEHP, and Σ phthalates was each associated with an increased likelihood of offspring ASD diagnosis at 4 years (AOR 1.89, 95% CI (1.01, 3.53), *p* = 0.05; AOR 1.55, 95% CI (1.06, 2.28), *p* = 0.03; AOR 1.55, 95% CI (1.00, 2.40), *p* = 0.05; respectively) and ASD traits at 4 years (AOR 1.44, 95% CI (1.03, 2.03), *p* = 0.04; AOR 1.51, 95% CI (1.15, 1.98), *p* = 0.003; AOR 1.55, 95% CI (1.20, 2.01), *p* = 0.0009; respectively) [31]. No associations were found between any phthalate daily intake levels and cognition at 2–3 years [31].

### 3.2. Association between gPFS^ox^ and Neurodevelopmental Outcomes 

Adjusting for age and sex, a higher gPFS^ox^ was associated with a greater likelihood of offspring ASD diagnosis and parent-reported ASD traits (Table 3). Each unit increase in gPFS^ox^, indicating the presence of two additional pro-oxidant alleles for genes in the oxidative stress response pathway, was associated with an AOR of 1.42 (95% CI (1.02, 2.01), *p* = 0.041) for ASD traits and 2.10 (95% CI (1.12, 4.11), *p* = 0.024) for ASD diagnosis. The associations (for log-odds) showed no significant departure from linearity. Associations for cognition, high-CBCL attention-deficit hyperactivity problems, and above-median CBCL autism spectrum problems did not reach significance (Table 3).

### 3.3. Interplay between gPFS^ox^ and Prenatal Phthalate Exposure against Neurodevelopmental Outcomes

**Figure 3:**
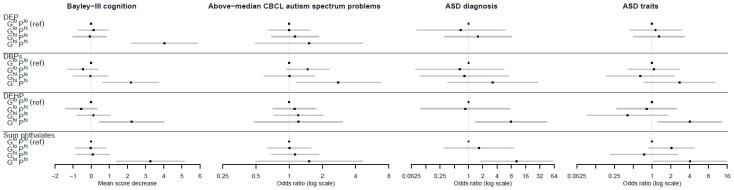
The association between the genetic pathway function score for oxidative stress response (gPFS^ox^) and prenatal phthalate level combinations and neurodevelopment. Note: Error bars are 95% confidence intervals; G^hi^ = top quintile of gPFS^ox^; G^lo^ = bottom four quintiles of gPFS^ox^; P^hi^ = top quintile of phthalate exposure; P^lo^ = bottom four quintiles of phthalate exposure; DEP = diethyl phthalate; DBPs = di-n-butyl phthalate (DnBP) + diisobutyl phthalate (DiBP); DEHP = di-(2-ethyl-5-oxohexyl) phthalate; sum phthalates = sum of DEP, DBPs, and DEHP.

To assess combined effects, gPFS^ox^ and prenatal maternal phthalate levels were each dichotomized as high (top quintile) vs. low (bottom four quintiles). The reference category was designated as individuals with both low gPFS^ox^ and low phthalate levels, denoted by G^lo^P^lo^. A clear pattern emerged (Figure 3, Table 4 and Table 5) in which individuals in the doubly exposed G^hi^P^hi^ group, but not other combinations, were at significantly higher risk of adverse neurodevelopment. For instance, for DEP and Bayley’s cognition, the estimated difference in mean score was 0.00 for the reference category; −1.2, *p* = 0.266 for G^lo^P^hi^; 0.09, *p* = 0.852 for G^hi^P^lo^; and −4.04, *p* < 0.0001 for G^hi^P^hi^. For DEHP and ASD diagnoses, the adjusted odds ratios were 1.00 for the reference category; 0.85, *p* = 0.884 for G^lo^P^hi^; insufficient numbers for G^hi^P^lo^; and 7.84, *p* = 0.019 for G^hi^P^hi^. Relative excess risk due to interaction (RERI) values were not significant, but using the attributable proportion (AP) metric, there was strong evidence of interactions for multiple phthalates across cognition, ASD diagnosis, and ASD traits outcomes. For example, the AP for DEHP and ASD diagnosis was 0.89 (95% CI (0.62, 1.16); *p* < 0.0001), indicating that the proportion of disease in the doubly exposed group (G^hi^P^hi^) due to interaction was 89%. The results for CBCL attention-deficit hyperactivity problems showed similar patterns (see Table S1).

## 4. Discussion

In this study, we employed a genetic pathway function score, gPFS^ox^, to capture genetic predisposition to oxidative stress based on transcriptional activity of the oxidative stress response pathway in multiple tissue types, including the brain. Using DNA samples sourced from cord and infant blood, we found that higher genetic scores for oxidative stress vulnerability were associated with ASD and autistic traits at 4 years of age. Unlike in our previous work, gene-environment interaction was evident, with children with both higher phthalate levels and a higher gPFS^ox^ (top quintile for both) having excess additive risk for ASD diagnosis or ASD traits at age 4 years. These findings expand our previous work with the use here of an externally derived score independent of disease status. 

The human brain has high energy demands and is vulnerable to oxidative damage [66]. Although oxidative damage in the adult brain has been well studied [67], the importance of oxidative stress in adverse brain development is being increasingly recognized. These findings support past work indicating that impaired redox control appears to be an underlying mechanism for gene–environment interactions in ASD [68,69], as both ASD-associated genes [37] and environmental risk factors of ASD have been associated with oxidative stress. Specifically regarding plastic chemicals, DBP metabolites have been associated with prenatal markers of oxidative stress [70]. Similarly, oxidative stress markers have been found to mediate the effect of higher DEHP levels on preterm birth [33]. 

Our findings are consistent with previous genetic studies linking oxidative stress to ASD etiology [31,39]. However, unlike existing genetic scores for oxidative stress, this functional, disease-agnostic approach provides clearer evidence that elevated ROS, independent of other factors, is at play. Evidence for an association with ASD at the genetic level also suggests that oxidative stress is causally involved rather than merely a byproduct of disrupted neurodevelopment, and importantly it reduces the likelihood of confounding by environmental factors. 

Our findings highlight the interplay between genetic susceptibility to oxidative stress and prenatal phthalate exposure. Individuals in the top quintile for gPFS^ox^ and top quintile for prenatal phthalate exposure, but generally not other combinations, were consistently at significantly higher risk of adverse neurodevelopment compared to those in the lower four quintiles for both. For doctor-diagnosed and parent-reported autism outcomes at 4 years, the combined top-quintile effect was evident for prenatal exposure to the high-molecular-weight phthalate DEHP and for Σ phthalates, a measure that includes DEHP, suggesting that DEHP may drive the association with ASD. This possibility is further supported by metabolomic analyses, with the association between higher prenatal DEHP exposure and increased ASD symptomology partly mediated through metabolic shifts closely linked to oxidative stress [71]. Testing for interaction, we found that the majority of the disease occurring in these doubly exposed groups was attributable to a gene–environment interaction. For example, the attributable proportion for the combined top quintiles of gPFS^ox^ and DEHP against ASD diagnosis due to interaction was more than 80 percent. These results for combined exposures are intuitively plausible given that the deleterious impact of impaired ROS processing—as reflected in a high gPFS^ox^—would be accentuated by any additional sources of ROS, such as phthalate exposure [33]. The results also highlight the risk posed to one-fifth of the population (i.e., individuals in the top quintile of gPFS^ox^) by phthalates and likely other common ROS-producing environmental pollutants. 

A key strength of this study is the improved interpretability of the genetic score. Here, gPFS^ox^ was derived from SNPs linked to expression levels of genes in the oxidative stress response pathway—encoding the logic of pro- and antioxidant genes acting in opposing directions—rather than to a disease or other outcome. This functional approach helps shed light on how the mechanism of oxidative stress response, including in brain tissue, relates to child cognition and ASD. Furthermore, gPFS^ox^ was constructed independently of the study cohort, using genotyping and RNA sequencing data from the GTEx consortium [61], thereby improving its potential to generalize to other study populations. Given that the score is disease-agnostic, gPFS^ox^ could be evaluated against other outcomes beyond early life hypothesized to involve oxidative stress, for instance neurodegenerative disorders such as Alzheimer’s disease, Parkinson’s disease, and amyotrophic lateral sclerosis [72]. Additional strengths of the study include comprehensive data and a range of neurodevelopmental outcomes from ages 2 to 4 years [31].

The study has several limitations. The number of doctor-diagnosed ASD cases was small and only 82% were verified by medical records. The autism problem score at 2 years was based on parental report, but we have previously demonstrated that this ASP score had an area under the curve of 0.93 (95% CI (0.82, 1.00)) for verified ASD diagnosis at age 4 years [65]. 

Here we only studied one environmental factor with possible oxidant action, phthalate plastic chemicals, but we have shown that other environmental factors such as prenatal maternal smoking or a lack of fish oil consumption (a putative antioxidant) may also be relevant [31]. Indeed, interpreted in the context of Mendelian randomization, where genetic data are used as proxies for environmental exposures, the findings for gPFS^ox^ itself suggest that wider oxidant exposures are important to consider. We plan to assess the level of agreement between the infant gPFS^ox^ and infant urinary oxidative markers such as 8-isopostane and 8-hydroxydeoxyguanosine [73,74] when these become available. Overall, our findings indicate that future studies should examine oxidant burden from both composite environmental and composite genetic factors as comprehensively as possible. This may provide additional clarity as to the possible adverse role of phthalate chemicals in neurodevelopment [24]. 

It is worth noting that gPFS^ox^ was constructed from a minimalist pathway of 12 genes, and several of these genes, such as *SP1*, exert pleiotropic effects across multiple signaling pathways. Despite this, by combining these genes in a shared pathway and encoding their cumulative influence on ROS, we found consistent effects that accord with past work on phthalates, oxidative stress, and neurodevelopment. However, future iterations could employ expanded pathways that capture a more comprehensive picture of the complex mechanisms underlying ROS balance. Moreover, while we aimed to generate a score with relevance to multiple disorders and therefore selected SNPs with the strongest overall effect on the expression of each gene, brain-region- or cell-type-specific variants of the score could be developed to study more localized pathophysiology. 

## 5. Conclusions

A novel genetic pathway function score for oxidative stress (gPFS^ox^), capturing transcriptional activity of the oxidative stress response pathway, has provided a genetic marker of oxidative stress that associates with adverse neurodevelopmental outcomes. Unlike our previous score for oxidative stress, gPFS^ox^ was constructed independently of our study cohort and has no a priori links to the neurodevelopmental conditions assessed here, improving its potential to generalize to other populations and to other ROS-related traits and disorders, as well as providing more robust evidence for the associations described. We also extended our previous work by using this improved functional genetic score to demonstrate that prenatal phthalate-induced adverse neurodevelopment will vary by host genetic oxidative stress vulnerability status. Future work on the causation of these neurodevelopmental disorders is likely to benefit from examining both environmental and genetic factors in the context of shared biological mechanisms.

## Figures and Tables

**Table 1:** Single-nucleotide polymorphisms (SNPs) used to create a genetic pathway function score for oxidative stress response (gPFS^ox^). SNPs were identified using the GTEx database as expression quantitative trait loci (eQTLs) for genes within this pathway.

	Gene	eQTL SNP	Effect of SNP on Gene Expression	Inferred Effect of SNP on Oxidative Stress
Pro-oxidant genes	*XDH*	rs45498201	increase	pro-oxidant
	*NOX4*	rs10830296	reduction	antioxidant
	*NFIX*	rs149677133	reduction	antioxidant
	*CYP1A1*	rs2470890	increase	pro-oxidant
Antioxidant genes	*MAPK10*	rs80320648	reduce	pro-oxidant
	*NFKB1*	rs28573147	increase	antioxidant
	*SP1*	rs35437931	increase	antioxidant
	*FOS*	rs79713290	increase	antioxidant
	*CAT*	rs12793666	increase	antioxidant
	*GPX1*	rs17650792	reduction	pro-oxidant
	*SOD1*	rs4998557	increase	antioxidant
	*SOD2*	rs5746105	increase	antioxidant

Note: The score for each participant was computed as the (unweighted) sum of pro-oxidant alleles they carry across all 12 SNPs.

**Table 2:** The distribution of key characteristics in the Barwon Infant Study.

	Full Cohort (N = 1074)			Participants with Any Neurodevelopment Data (N = 868)		
	N	Mean (SD) or GM {GSD} or % [n]		N	Mean (SD) or GM {GSD} or % [n]	
**Genotype**						
Oxidative stress genetic score	1031	6.8	(1.0)	850	6.8	(1.0)
**Phthalate daily intakes (µg/kg bw/day)**						
DEP daily intake	847	1.6	{3.8}	756	1.6	{3.8}
DBPs daily intake	847	1.9	{2.0}	756	1.9	{2.0}
DEHP (oxidized) daily intake	847	1.6	{2.1}	756	1.6	{2.1}
Σ phthalate daily intake	847	6.3	{2.2}	756	6.3	{2.2}
DEP daily intake top quintile (≥4.459)	847	20.2%	[171]	756	20.8%	[157]
DBPs daily intake top quintile (≥3.362)	847	20.1%	[170]	756	20.5%	[155]
DEHP (oxidized) daily intake top quintile (≥2.614)	847	20.1%	[170]	756	19.8%	[150]
Σ phthalate daily intake top quintile (≥11.043)	847	20.1%	[170]	756	20.4%	[154]
Gestational age at maternal urine collection (weeks)	847	36.3	(0.7)	756	36.3	(0.7)
**Demographic and household factors**						
Maternal age at conception (years)	1074	31.3	(4.8)	868	31.8	(4.5)
Paternal age at conception (years)	1024	33.5	(5.9)	830	33.8	(5.6)
British/European ancestry (all 4 grandparents)	1060	73.0%	[774]	861	73.8%	[635]
Maternal university-level education	1068	51.3%	[548]	865	55.8%	[483]
Parental marital status (married)	1071	70.4%	[754]	868	74.1%	[643]
Older siblings of child living at home (one or more)	1072	55.0%	[590]	865	55.2%	[478]
**Prenatal, perinatal, and postnatal factors**						
Gestational age at birth (weeks)	1074	39.4	(1.5)	868	39.4	(1.5)
Child sex at birth (male)	1074	51.7%	[555]	868	52.6%	[457]
**Child neurodevelopment**						
Bayley-III Cognitive Scale raw score	678	71.1	(4.1)			
Bayley-III Cognitive Scale scaled score	678	10.8	(2.1)			
Bayley-III Cognitive Scale raw score <70	678	34.7%	[235]			
Child age at Bayley-III assessment (months)	678	29.4	(1.7)			
CBCL autism spectrum problems (T-score above 50)	676	36.8%	[249]			
CBCL attention-deficit hyperactivity problems (T-score above 65)	676	5.2%	[35]			
Child age at CBCL assessment (months)	677	29.5	(1.8)			
Autism spectrum disorder doctor diagnosis	791	1.4%	[11]			
Parent-reported autistic traits	791	4.9%	[39]			
Child age at 4-year review (months)	791	49.9	(3.1)			

NB: SD = standard deviation; GM = geometric mean; GSD = geometric standard deviation; bw = body weight; DEP = diethyl phthalate; DBPs = di-n-butyl phthalate (DnBP) + diisobutyl phthalate (DiBP); DEHP = di-(2-ethyl-5-oxohexyl) phthalate; Σ phthalates = sum of DEP, DBPs, and DEHP; CBCL = Child Behavior Checklist for Ages 1.5–5.

**Table 3:** Main effect of genetic pathway function score for oxidative stress response (gPFS^ox^) against cognitive and ASD outcomes.

	Bayley-III Cognition		Above-Median CBCL Autism Spectrum Problems				ASD Diagnosis				ASD Traits			
	Adj. Mean Difference (95% CI)	*p* Value	YES: Mean (SD) or % [n]	NO: Mean (SD) or % [n]	AOR (95% CI)	*p* Value	YES: Mean (SD) or % [n]	NO: Mean (SD) or % [n]	AOR (95% CI)	*p* Value	YES: Mean (SD) or % [n]	NO: Mean (SD) or % [n]	AOR (95% CI)	*p* Value
gPFS^ox^ (Per 2 additional pro-oxidant alleles)	−0.20 (−0.50, 0.10)	0.197	6.8 (1.0)	6.7 (0.9)	1.10 (0.94, 1.30)	0.250	7.5 (1.2)	6.8 (1.0)	2.10 (1.12, 4.11)	0.024	7.1 (0.9)	6.8 (1.0)	1.42 (1.02, 2.01)	0.041
gPFS^ox^ (Top quintile vs. rest)	−0.49 (−1.31, 0.29)	0.231	17.0% [41]	14.8% [62]	1.11 (0.68, 1.71)	0.637	36.4% [4]	17.7% [135]	2.56 (0.74, 9.03)	0.140	21.1% [8]	17.8% [131]	1.20 (0.54, 2.67)	0.654

Note: There were no associations between the phthalate exposures and gPFS^ox^. AOR = adjusted odds ratio. For Bayley’s cognition, models were adjusted for post-conceptional age at test, sex, administering researcher, and experience of researcher in test administration. For above-median CBCL autism spectrum problems, ASD diagnosis, and ASD traits, models were adjusted for age and sex.

**Table 4:** Distributions of neurodevelopmental outcomes by genetic pathway function score for oxidative stress response (gPFS^ox^) and prenatal phthalate level combinations.

		Bayley-III Cognition			Above-Median CBCL Autism Spectrum Problems		ASD Diagnosis		ASD Traits	
	Gene-Phthalate Subgroup	N	Mean (SD)	% [n] ^1^	N	% [n] ^2^	N	% [n] ^3^	N	% [n] ^4^
DEP	G^lo^P^lo^	396	71.4 (3.9)	32.6 [129]	391	36.1 [141]	438	1.4 [6]	438	5.0 [22]
	G^lo^P^hi^	110	71.2 (4.2)	31.8 [35]	111	36.0 [40]	120	0.8 [1]	120	5.0 [6]
	G^hi^P^lo^	82	71.6 (3.9)	29.3 [24]	82	39.0 [32]	100	2.0 [2]	100	6.0 [6]
	G^hi^P^hi^	18	67.6 (7.6)	72.2 [13]	13	46.2 [6]	19	0.0 [0]	19	0.0 [0]
DBPs	G^lo^P^lo^	403	71.2 (4.1)	34.0 [137]	398	34.2 [136]	444	1.4 [6]	444	5.0 [22]
	G^lo^P^hi^	103	71.7 (3.3)	26.2 [27]	104	43.3 [45]	114	0.9 [1]	114	5.3 [6]
	G^hi^P^lo^	74	71.5 (4)	32.4 [24]	73	34.2 [25]	91	1.1 [1]	91	3.3 [3]
	G^hi^P^hi^	26	69.3 (7)	50.0 [13]	22	59.1 [13]	28	3.6 [1]	28	10.7 [3]
DEHP	G^lo^P^lo^	408	71.1 (3.9)	34.6 [141]	397	35.5 [141]	447	1.3 [6]	447	5.4 [24]
	G^lo^P^hi^	98	72.1 (3.8)	23.5 [23]	105	38.1 [40]	111	0.9 [1]	111	3.6 [4]
	G^hi^P^lo^	81	71.3 (4.1)	35.8 [29]	75	40.0 [30]	91	0.0 [0]	91	2.2 [2]
	G^hi^P^hi^	19	69.3 (7.7)	42.1 [8]	20	40.0 [8]	28	7.1 [2]	28	14.3 [4]
Σ phthalates	G^lo^P^lo^	401	71.3 (3.9)	33.7 [135]	397	36.0 [143]	440	1.1 [5]	440	4.3 [19]
	G^lo^P^hi^	105	71.6 (4)	27.6 [29]	105	36.2 [38]	118	1.7 [2]	118	7.6 [9]
	G^hi^P^lo^	83	71.5 (4.1)	32.5 [27]	82	39.0 [32]	97	0.0 [0]	97	3.1 [3]
	G^hi^P^hi^	17	68.2 (7.7)	58.8 [10]	13	46.2 [6]	22	9.1 [2]	22	13.6 [3]

^1^ A raw score below 70 on the Bayley-III Cognitive scale administered at 2 years of age. ^2^ A T-score above 50 on the CBCL autism spectrum problems (ASP) scale administered at 2 years of age. ^3^ Parent-reported autism spectrum disorder (ASD) diagnosis at 4 years of age. ^4^ Parent-reported autistic traits at 4 years of age. G^hi^ = top quintile of gPFS^ox^; G^lo^ = bottom four quintiles of gPFS^ox^; P^hi^ = top quintile of phthalate exposure; P^lo^ = bottom four quintiles of phthalate exposure; DEP = diethyl phthalate; DBPs = di-n-butyl phthalate (DnBP) + diisobutyl phthalate (DiBP); DEHP = di-(2-ethyl-5-oxohexyl) phthalate; Σ phthalates = sum of DEP, DBPs, and DEHP.

**Table 5:** Interplay on the additive scale between genetic pathway function score for oxidative stress response (gPFS^ox^) and prenatal phthalate levels against neurodevelopmental outcomes.

	G	P	G^lo^ P^hi^		G^hi^ P^lo^		G^hi^ P^hi^		Additive Interaction	
	gPFS^ox^	Phthalate								
			Adj Mean Difference (95% CI)	*p* Value	Adj Mean Difference (95% CI)	*p* Value	Adj Mean Difference (95% CI)	*p* Value	AP (95% CI)	*p* Value
**Bayley-III cognition ^a^**		DEP	−0.12 (−0.93, 0.69)	0.766	0.09 (−0.83, 1.00)	0.852	**−4.04 (−5.85, −2.22)**	**<0.0001**	**0.82 (0.62, 1.01)**	**<0.0001**
		DBPs	0.46 (−0.37, 1.30)	0.276	0.04 (−0.91, 1.00)	0.927	**−2.19 (−3.72, −0.66)**	**0.005**	**0.71 (0.43, 1.03)**	**<0.0001**
		DEHP	0.55 (−0.32, 1.42)	0.212	−0.12 (−1.04, 0.80)	0.796	**−2.23 (−4.01, −0.45)**	**0.014**	0.30 (−0.62, 1.16)	0.511
		Σ phthalates	0.04 (−0.79, 0.88)	0.922	−0.08 (−1.00, 0.83)	0.856	**−3.26 (−5.13, −1.39)**	**0.001**	**0.72 (0.39, 1.10)**	**<0.0001**
			**AOR (95% CI)**	***p* value**	**AOR (95% CI)**	***p* value**	**AOR (95% CI)**	***p* value**		
**Above-median** **CBCL autism spectrum problems**		DEP	1.00 (0.64, 1.55)	0.993	1.13 (0.69, 1.85)	0.615	1.51 (0.50, 4.62)	0.468	0.04 (−1.21, 1.34)	0.952
		DBPs	1.47 (0.95, 2.28)	0.086	1.00 (0.59, 1.70)	0.986	**2.78 (1.16, 6.69)**	**0.022**	0.43 (−0.17, 1.03)	0.146
		DEHP	1.12 (0.72, 1.75)	0.625	1.21 (0.73, 2.01)	0.465	1.21 (0.48, 3.04)	0.682	−0.10 (−1.28, 1.14)	0.874
		Σ phthalates	1.01 (0.64, 1.58)	0.977	1.13 (0.70, 1.85)	0.613	1.52 (0.50, 4.63)	0.46	0.28 (−0.61, 1.24)	0.554
**ASD diagnosis**		DEP	0.69 (0.08, 5.83)	0.729	1.58 (0.31, 8.02)	0.583	---		−0.26 (−3.48, 2.91)	0.872
		DBPs	0.65 (0.08, 5.49)	0.693	0.82 (0.10, 6.93)	0.854	3.23 (0.37, 28.61)	0.291	**0.86 (0.08, 1.62)**	**0.025**
		DEHP	0.85 (0.88, 0.10)	0.884	---		**7.84 (1.40, 43.98)**	**0.019**	**0.89 (0.62, 1.16)**	**<0.0001**
		Σ phthalates	1.65 (0.31, 8.70)	0.554	---		**10.24 (1.76, 59.48)**	**0.01**	**0.84 (0.51, 1.17)**	**<0.0001**
**ASD traits**		DEP	1.14 (0.44, 2.90)	0.791	1.30 (0.51, 3.36)	0.582	---		−0.44 (−0.24, 1.36)	0.632
		DBPs	1.07 (0.42, 2.75)	0.881	0.65 (0.19, 2.26)	0.502	2.77 (0.71, 10.11)	0.122	**0.74 (0.21, 1.25)**	**0.009**
		DEHP	0.82 (0.27, 2.46)	0.724	0.40 (0.09, 1.76)	0.226	**4.01 (1.23, 13.01)**	**0.021**	**0.94 (0.66, 1.22)**	**<0.0001**
		Σ phthalates	2.04 (0.88, 4.70)	0.095	0.75 (0.21, 2.60)	0.645	**4.06 (1.06, 15.61)**	**0.041**	0.56 (−0.13, 1.26)	0.114

Note: Each combination of G and P is compared against the reference category G^lo^P^lo^; “---” indicates insufficient outcome numbers in category. For Bayley’s cognition, models were adjusted for post-conceptional age at test, sex, administering researcher, and experience of researcher in test administration. For above-median CBCL autism spectrum problems, ASD diagnosis, and ASD traits, models were adjusted for post-conceptional age at testing and sex. G^hi^ = top quintile of gPFS^ox^; G^lo^ = bottom four quintiles of gPFS^ox^; P^hi^ = top quintile of phthalate exposure; P^lo^ = bottom four quintiles of phthalate exposure; AP = attributable proportion, or proportion of disease in doubly exposed group due to interaction; AOR = adjusted odds ratio; DEP = diethyl phthalate; DBPs = di-n-butyl phthalate (DnBP) + diisobutyl phthalate (DiBP); DEHP = di-(2-ethyl-5-oxohexyl) phthalate; Σ phthalates = sum of DEP, DBPs, and DEHP. ^a^ For additive interaction, Bayley’s cognition variable was dichotomized using clinical cutoff of cognition <70 (at least moderate cognitive deficit).

## Data Availability

For laboratory data access contact the corresponding author. Access to BIS data including all data used in this paper can be requested through the BIS Steering Committee by contacting annelouise.ponsonby@florey.edu.au. Requests to access cohort data are considered on scientific and ethical grounds and, if approved, provided under collaborative research agreements. Additional project information, including cohort data description and access procedure, is available at the project’s website https://www.barwoninfantstudy.org.au (last accessed 28 March 2022).

## Supplementary Materials

The following supporting information can be downloaded at: https://www.mdpi.com/article/10.3390/antiox11040659/s1, Table S1: Interplay on the additive scale between the genetic Pathway Function Score for oxidative stress response (gPFS^ox^) and prenatal phthalate levels against borderline/clinical attention-deficit hyperactivity problems.
