# Supplementary material for: A Pathway-Based Genetic Score for Oxidative Stress: An Indicator of Host Vulnerability to Phthalate-Associated Adverse Neurodevelopment
Source: Antioxidants (Basel). 2022 Mar 29;11(4):659. doi: 10.3390/antiox11040659 (PMC9030597; doi:10.3390/antiox11040659)
Supplement: Supplementary file 1 [file antioxidants-11-00659-s001.zip › antioxidants-1619686-supplementary.pdf]

Supplemental Material

A pathway-based genetic score for oxidative stress: an indicator of host vulnerability to phthalate-associated adverse neurodevelopment.

Table S1: Interplay on the additive scale between the genetic Pathway Function Score for oxidative stress response ($gPFS^{ox}$) and prenatal phthalate levels against borderline/clinical attention-deficit hyperactivity problems.

**G** **$gPFS^{ox}$**	**P** **Phthalate**	**G^{lo} P^{hi}**		**G^{hi} P^{lo}**		**G^{hi} P^{hi}**		**Additive interaction**	
		**AOR (95% CI)**	***p* value**	**AOR (95% CI)**	***p* value**	**AOR (95% CI)**	***p* value**	**AP (95% CI)**	***p* value**
**High CBCL attention-deficit hyperactivity problems**	DEP	1.19 (0.71, 2.01)	0.503	1.28 (0.75, 2.20)	0.362	**2.86 (1.10, 7.45)**	**0.031**	0.48 (-0.08, 1.05)	0.092
	DBPs	0.89 (0.51, 1.55)	0.684	1.34 (0.78, 2.31)	0.291	1.63 (0.69, 3.86)	0.268	0.24 (-0.56, 1.05)	0.552
	DEHP	0.61 (0.33, 1.13)	0.115	1.17 (0.68, 2.03)	0.565	1.87 (0.80, 4.36)	0.146	**0.58 (0.07, 1.09)**	**0.025**
	Σ phthalates	1.07 (0.63, 1.81)	0.809	1.18 (0.68, 2.04)	0.560	**3.05 (1.25, 7.42)**	**0.014**	**0.59 (0.15, 1.03)**	**0.008**

Note. Each combination of G and P is compared against the reference category $G^{lo}P^{lo}$. “High CBCL attention-deficit hyperactivity problems” was defined using a T-score cutoff of above 65 for CBCL attention-deficit hyperactivity problems, indicating “borderline or clinical ADHD symptoms” (see methods). The model was adjusted for post-conceptional age at test and sex. G^{hi} = top quintile of $gPFS^{ox}$; G^{lo} = bottom four quintiles of $gPFS^{ox}$; P^{hi} = top quintile of phthalate exposure; P^{lo} = bottom four quintiles of phthalate exposure; AP = attributable proportion, or proportion of disease in doubly exposed group due to interaction; AOR = adjusted odds ratio; DEP = diethyl phthalate; DBP = di-n-butyl phthalate (DnBP) + diisobutyl phthalate (DiBP); DEHP = di-(2-ethyl-5-oxohexyl) phthalate; Σ phthalates = sum of DEP, DBPs, and DEHP.
